# Leader- and Terminal Residue Requirements for Circularin A Biosynthesis Probed by Systematic Mutational Analyses

**DOI:** 10.1021/acssynbio.2c00661

**Published:** 2023-03-01

**Authors:** Fangfang Liu, Auke J. van Heel, Oscar P. Kuipers

**Affiliations:** Department of Molecular Genetics, Groningen Biomolecular Sciences and Biotechnology Institute, University of Groningen, 9747 AG Groningen, The Netherlands

**Keywords:** circularin A, biosynthesis, antimicrobial activity, mutational analysis, leader peptide, terminal residues

## Abstract

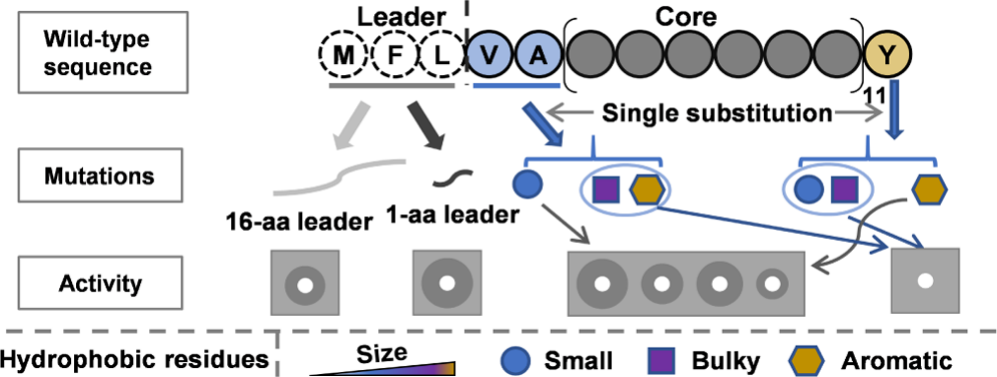

Circularin A is a circular bacteriocin belonging to a subgroup of the ribosomally synthesized and post-translationally modified peptide (RiPP) superfamily. The post-translational biosynthesis of circular bacteriocins primarily consists of leader cleavage, core peptide circularization, and bacteriocin secretion. However, none of these processes have been fully elucidated due to the complex biosynthesis of such bacteriocins and the lack of homology to the functions of other known biosynthetic enzymes. In this study, we investigated the leader- and terminal residue requirements for the biosynthesis of circularin A by systematic mutational analyses, including the mutational effects of variable leader lengths, as well as site-directed substitutions of residues at positions near the leader cleavage site and the circularization site. Results show that the leader with only one Met residue, the shortest leader possible, is sufficient to produce mature circularin A; helix-forming short-sidechain hydrophobic residues are required at positions Val1 and Ala2 of the N-terminus to form active peptide derivatives, indicating the possible steric hindrance effect at these two positions; and an aromatic residue is required at the C-terminal Tyr69 position to produce a mature circular derivative. However, the requirements for residues at position Ala68 are much more relaxed relative to the positions of Val1 and Ala2, since even substitution with the largest possible residue, i.e., tryptophan, still allows the generation of an active Ala68Trp derivative. Our findings provide new perspectives for the biosynthesis of this short-leader circular bacteriocin, which enables the application of circular bacteriocin biosynthesis in rational modified peptide engineering.

## Introduction

Circular bacteriocins are a group of antimicrobial peptides (AMPs), which belong to a characteristic subgroup of ribosomally synthesized and post-translationally modified peptides (RiPPs). The term “circular” indicates that the backbone of these peptides is characterized by head-to-tail ligation, differentiating it from other cyclic peptides that contain oxazole/thiazole or lanthionine rings and/or disulfide bridges. Until now, there are 21 circular bacteriocins that have been characterized to some extent. It has been suggested to divide circular bacteriocins into two major subgroups mainly based on their biochemical characteristics such as peptide hydrophobicity, net charge, and isoelectric point. The most recent review listing the 14 reported circular bacteriocins was written in 2018.^1^ Since new circular bacteriocins have been discovered in the past few years, we include here an updated list of 21 characterized circular bacteriocins in Table 1.

**Table 1:** Characteristics of Circular Bacteriocins

bacteriocin	leader peptide (aa)	mature peptide (aa)	MW^a^ (Da)	pI^b^	net charge^b^	hydrophobicity (GRAVY)^b^	producer strain	reference
Group I with a Short Leader								
circularin A	3	69	6770.05	10.46	+4	1.007	*Clostridium beijerinckii* ATCC 25752	(2)
uberolysin	6	70	7048.30	9.60	+3	0.937	*Streptococcus uberis* 42	(3)
carnocyclin A	4	60	5862.05	10.00	+4	1.058	*Carnobacterium maltaromaticum* UAL307	(4)
lactocyclicin Q	2	61	6060.16	9.70	+4	0.826	*Lactococcus* sp. QU 12	(5)
leucocyclicin Q	2	61	6115.23	9.53	+3	0.744	*Leuconostoc mesenteroides* TK41401	(6)
garvicin ML	3	60	6007.27	10.13	+5	0.887	*Lc. garvieae* DCC43	(7)
cerecyclin	4	70	7071.39	10.00	+4	0.563	*Bacillus cereus* DDD103	(8)
bacicyclicin XIN-1	4	60	5851.93	10.29	+3	0.877	*Bacillus*. sp. Xin1	(9)
aureocyclicin_4185	4	60	5607.65	10.00	+3	0.973	*Staphylococcus aureus* 4185	(10)
Group I with a Long Leader								
enterocin AS-48	35	70	7149.56	10.09	+6	0.539	*Enterococcus faecalis* S-48	(11)
amylocyclicin	48	64	6381.62	9.82	+5	0.850	*Bacillus amyloliquefaciens* FZB42	(12)
enterocin NKR-5-3B	23	64	6316.53	9.90	+5	0.953	*Enterococcus faecium* NKR-5-3	(13)
pumilarin	38	70	7187.42	10.00	+5	0.579	*Bacillus pumilus* B4107	(14)
BacA	35	70	7149.56	10.09	+6	0.539	*Enterococcus faecalis* plasmid pPD1	(15)
amylocyclicin_CMW1	47	64	6351.59	9.82	+5	0.889	*Bacillus amyloliquefaciens* CMW1	(16)
Group II								
gassericin A	33	58	5653.60	6.75	+1	0.997	*Lactobacillus gasseri* LA39	(17)
butyrivibriocin AR10	22	58	5981.96	4.03	–2	1.002	*Butyrivibrio fibrisolvens* AR10	(18)
acidocin B	33	58	5621.54	6.75	+1	1.036	*Lb. acidophilus* M46	(19)
plantaricyclin A	33	58	5570.53	8.60	+2	1.057	*Lb. plantarum* NI326	(20)
plantacyclin_B21AG	33	58	5667.70	9.99	+3	1.002	*Lb. plantarum* B21	(21)
paracyclicin	24	58	5906.90	6.74	+1	1.003	*Lb. paracesei* DSM 5622	(22)

a Predicted for the circular form of the bacteriocin using Expasy ProtParamTool.

b Predicted for the linear form of the bacteriocin without the leader using Expasy ProtParamTool. GRAVY: grand average of hydropathicity.

From the list, we can easily notice that the leader lengths of circular bacteriocins are quite variable, ranging from 2 to 48 amino acids (aa). All six members in subgroup II have relatively long leader sequences and relatively low isoelectric points (pI) and net charges. In contrast, subgroup I circular bacteriocins have relatively high isoelectric points (pI) and net charges, and the leader lengths vary significantly. In total, there are nine circular bacteriocins reported with short leaders, which are circularin_A (leader sequence: MFL), cerecyclin (leader sequence: MLFN), uberolysin (leader sequence: MDILLE), lactocyclicin_Q (leader sequence: MK), leucocyclicin_Q (leader sequence: MF), garvicin_ML (leader sequence: MFD), carnocyclin_A (leader sequence: MLYE), bacicyclicin XIN-1 (leader sequence: MLFE), and aureocyclicin 4185 (leader sequence: MLLE).

The leader peptides of bacteriocins classified in the same group often share a common motif that plays a role in the substrate–enzyme interaction processes,^23^ such as the conserved FNLD box in leader peptides of class I lantibiotic bacteriocins^24^ and the highly conserved double glycine motif (GG) in leader peptides of class II bacteriocins.^25,26^ However, the leader peptides of circular bacteriocins vary considerably in both their lengths and amino acid sequences, making it hard to predict their functions in bacteriocin biosynthesis.^1^ Previous mutational studies in the leader peptide of enterocin Ent53B have shown that leader truncations disabled bacteriocin production, while single-residue substitution mutants imposed a variable effect on bacteriocin production likely by reducing or enhancing its interaction with processing enzyme(s).^27^ Based on previous observations, it has been proposed that the leader peptides of circular bacteriocins are essential in bacteriocin biosynthesis, although they may not (solely) function as recognition signals for bacteriocin processing, i.e., leader removal, peptide circularization, and bacteriocin secretion.^1^

Circular bacteriocins are characterized by head-to-tail ligation, resulting in a peptide bond between the N- and C-terminal residues of the core peptide.^28^ Despite the low similarity in primary peptide sequences (Figure S1), circular bacteriocins share a conserved compact structure consisting of 4–5 α-helices.^29^ The circularization site is located within a helical structure and is surrounded by hydrophobic residues. Mutational studies of Ent53B, a circular bacteriocin with a 23-aa leader, suggested that substitutions of Leu1 with helix-forming hydrophobic residues retained the production of mature Ent53B derivatives, whereas mutations with non-hydrophobic residues and helix-breaker residues failed to produce the mature bacteriocin.^27^ It would be interesting to also investigate the mutational effects of residues at the ligation site and nearby residues in other circular bacteriocins, especially the ones with short leaders.

To date, the biosynthetic mechanism of circular bacteriocins still remains poorly understood,^1^ i.e., leader cleavage, core peptide circularization, and bacteriocin secretion have not yet been fully elucidated. It is generally accepted that the products of essential genes in circular bacteriocin biosynthetic gene clusters probably form a protein complex to cooperatively mediate bacteriocin processing, since the production of (pre-)mature circular bacteriocins was blocked when knockouts of a single gene in their biosynthetic gene clusters were introduced.^30,31^ It was previously thought that these events of bacteriocin processing could be coupled reactions. Recently, more evidence has favored that they are independent reactions: leader cleavage and bacteriocin circularization have been reported to be separate processes in garvicin ML biosynthesis,^32^ and bacteriocin circularization and bacteriocin secretion have been reported to be independent processes in leucocyclicin Q biosynthesis.^33^ Understanding the mechanism of circular bacteriocin biosynthesis is arguably the most challenging part before realizing their enormous potential as antimicrobial compounds for industrial or medical applications. Mutagenesis offers a powerful approach to eventually elucidate the underlying complex post-translational modification machinery.

Previously, we successfully achieved functional production of clostridial circularin A in *Lactococcus lactis* NZ9000 by expressing the *cirABCDE* gene cluster using the convenient nisin-controlled expression (NICE) system.^34^ Within the cluster *cirABCDE*, *cirA* encodes the bacteriocin precursor, *cirBCD-*encoded proteins are responsible for circularin A maturation and secretion, and the product of *cirE* is a dedicated immunity protein that confers self-protection to its host strain. With this *L. lactis* production platform and the methodology developed previously, we here investigate the role of the 3-aa leader peptide in circularin A biosynthesis and the effects of residue substitutions at the ligation sites and nearby residues on its biosynthesis and bioactivity. Activity levels were assessed by colony overlay assays, and production yields were estimated by the band intensities in protein gels. Since activity assays are more sensitive and provide reliable and reproducible results, our findings are primarily presented in the form of activity results. For the leader mutants, activity levels are expected to reflect bacteriocin production yields since the core peptide sequence remains intact. However, mutations in the core peptide of circularin A may alter the delicate structure of the bacteriocin and lead to a discrepancy between the activity level and the peptide yield. These experiments contribute to a better understanding of circularin A production and our findings reveal new insights that significantly help to further elucidate the biosynthesis of circular bacteriocins.

## Results and Discussion

### Aromatic Residue F-2 in the Leader Peptide of Circularin A is More Important in Retaining Efficient Biosynthesis Than Residue L-1

Circularin A is a circular bacteriocin with a very short leader. The leader sequence consists of only three hydrophobic residues: Met-3, Phe-2, and Leu-1. To evaluate the effect of the leader sequence on bacteriocin production, site-directed mutagenesis was performed in the leader peptide of circularin A. Specifically, positions −1 (Leu, L) and −2 (Phe, F) were nonsimultaneously mutated into either a positively charged residue lysine (Lys, K) or a negatively charged residue aspartic acid (Asp, D) to investigate the effects of introducing charged residues into the leader peptide, creating four leader variants (MFK, MFD, MKL, and MDL). Additionally, another two mutants were investigated near the cleavage site to study their effects on leader cleavage, which included substitutions at position Leu-1 with either the short-sidechain hydrophobic residue Ala or the bulky sidechain hydrophobic residue Trp (leader variants MFA and MFW). Moreover, a leader variant MAA was also created with double alanine substitutions at positions −2 and −1. In total, seven leader mutants were made with residue substitutions in the leader peptide of circularin A, including MKL, MDL, MFK, MFD, MFA, MFW, and MAA.

**Figure 1:**
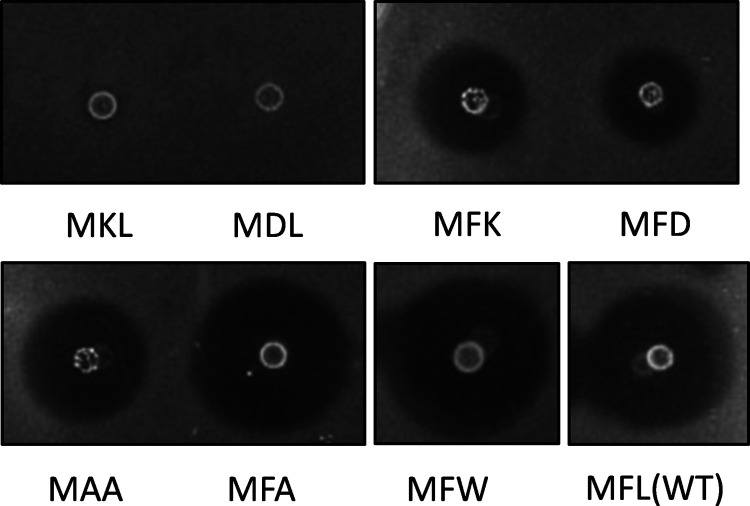
Antimicrobial activity of leader variants with site-directed substitutions. Indicator strain: *Lactobacillus sake* ATCC 15521.

The overlay activity tests showed that the charged residues in the leader of CirA generally decreased antimicrobial activity (Figure 1), especially for mutants with charged residues at position F-2 (leader variants MKL and MDL). The activities of leader mutants F-2K and F-2D were barely observed, while mutants L-1K and L-1D (MFK and MFD) retained certain levels of antimicrobial activity, indicating a better bacteriocin biosynthesis in L-1 mutants than their F-2 mutational counterparts (since the mature resulting peptides of all leader variants were expected to be identical once circularized, i.e., when leaders were cleaved off). Mutagenesis studies of the Ent53B leader also showed that M-1 mutants retained better activity than P-2 mutants when mutated to charged residues (D and E), with the relative activity of 85, 115, 0, and 54% for M-1E, M-1D, P-2E, and P-2D, respectively.^27^ Moreover, L-1 substitutions of CirA with hydrophobic residues (A and W) largely retained antimicrobial activity against the indicator strain *Lb. sake* ATCC 15521. Alanine is a hydrophobic residue with a rather short sidechain and tryptophan is the largest sidechain hydrophobic residue. However, the mutant L-1A (leader MFA) displayed comparable activity levels to the mutant L-1W (leader MFW), and both variants of MFA and MFW were slightly more active than the wild type. Substitutions at position M-1 in Ent53B showed variable effects, with relative activity of 77 and 100% for M-1A and M-1T, respectively; whereas mutants M-1V and M-1S are completely inactive.^27^ Mutagenesis of the AS-48 leader peptide revealed that H-1I disabled antimicrobial activity.^35^ It is worth noting that Ent53B and AS-48 are circular bacteriocins with long leaders, and thus their leader processing might differ significantly from the ones with short leaders.

For the leader variant with double alanine substitutions at the F-2 and L-1 positions, leader MAA retained considerable activity (Figure 1), ∼70% activity of the wild-type leader. However, comparing the activity levels between leader MFA and leader MAA, alanine substitution at position F-2 leads to a significant decrease in antimicrobial activity. The recognition site for leader cleavage is usually located in the last (few) residue(s) of the leader peptide, such as the ASPR sequence in nisin biosynthesis and the double glycine motif (GG) at −1 and −2 positions in class II bacteriocins.^36^ Here, we found in the leader peptide of circularin A that L-1 was more flexible to substitutions in retaining bacteriocin production, whereas mutants of the aromatic residue F-2 tended to dramatically decrease bacteriocin production, in particular, when charges were introduced.

The effects of leader mutants on antimicrobial activity were confirmed by matrix-assisted laser desorption/ionization-time-of-flight (MALDI-TOF) analysis. As stated earlier, the core peptide sequence remains intact for all leader variants, which means that the mature resulting peptides will be all identical if (correctly) circularized. As expected, MALDI-TOF spectrometry data confirmed that the active compound produced from various leader variants was fully modified bacteriocin circularin A (Figure S2). Moreover, the activity levels of leader variants are expected to reflect their bacteriocin yields since it has been shown that linear peptides of this family are devoid of activity.^32^ For the inactive leader mutants (e.g., MKL, MDL), we could not detect the target peptide, implying that leader cleavage was blocked in these mutants and thus circularization did not take place. It is well-known that the linear unmodified peptides could be easily degraded by intracellular peptidases/proteases when expressed in *L. lactis* (an example of (partial)degradation is described later in this study). The tricine-sodium dodecyl-sulfate polyacrylamide gel electrophoresis (tricine-SDS-PAGE) protein gels of leader variants are shown in Figure S3.

Overall, hydrophobic residues are more favored than charged residues in the leader of circularin A for bacteriocin biosynthetic processing and/or circularization. The interaction of the CirA precursor with its cognate enzyme(s) could be a hydrophobic-interaction-driven process although the electrostatic-interaction-driven process has been commonly reported for peptide (or protein) maturation.^37,38^ A similar phenomenon was also observed in Ent53B biosynthesis.^27^ In fact, we notice that all characterized circular bacteriocins have at least one of the charged residues or aromatic residues in their leader peptides, even the ones with short leaders. Among nine circular bacteriocins that have short leaders, the charged residues are often located at position −1 and aromatic residues at position −2, accounting for 66.67 and 55.56%, respectively (Table S1). These charged or aromatic residues may play an important role in their respective bacteriocin biosynthesis.

### Leader of Just One Amino Acid Residue (Met) is Sufficient to Generate Mature Circularin A

The leader lengths of circular bacteriocins vary significantly (Table 1). The shortest leader reported for bacteriocin biosynthesis, except for the known class of leaderless bacteriocins, consists of two amino acids (aa), which has been reported for Lactocyclicin_Q^5^ (leader sequence: MK) and Leucocyclicin_Q^6^ (leader sequence: MF). The huge differences in leader length and the lack of sequence homology make it difficult to predict their roles in the biosynthesis of circular bacteriocins. To investigate the effect of leader length on the bacteriocin production of circularin A, three truncated leader mutants (MF, ML, M) and three extended leader mutants including a 6xhis-tag (M_His6_, M_His6_FL, MFLVA-_His6_-GGMFL) were genetically constructed. The his-tag constructs also have the potential to facilitate intracellular peptide purification and immunological studies such as Western blotting, which will be further investigated in another study/paper.

**Figure 2:**
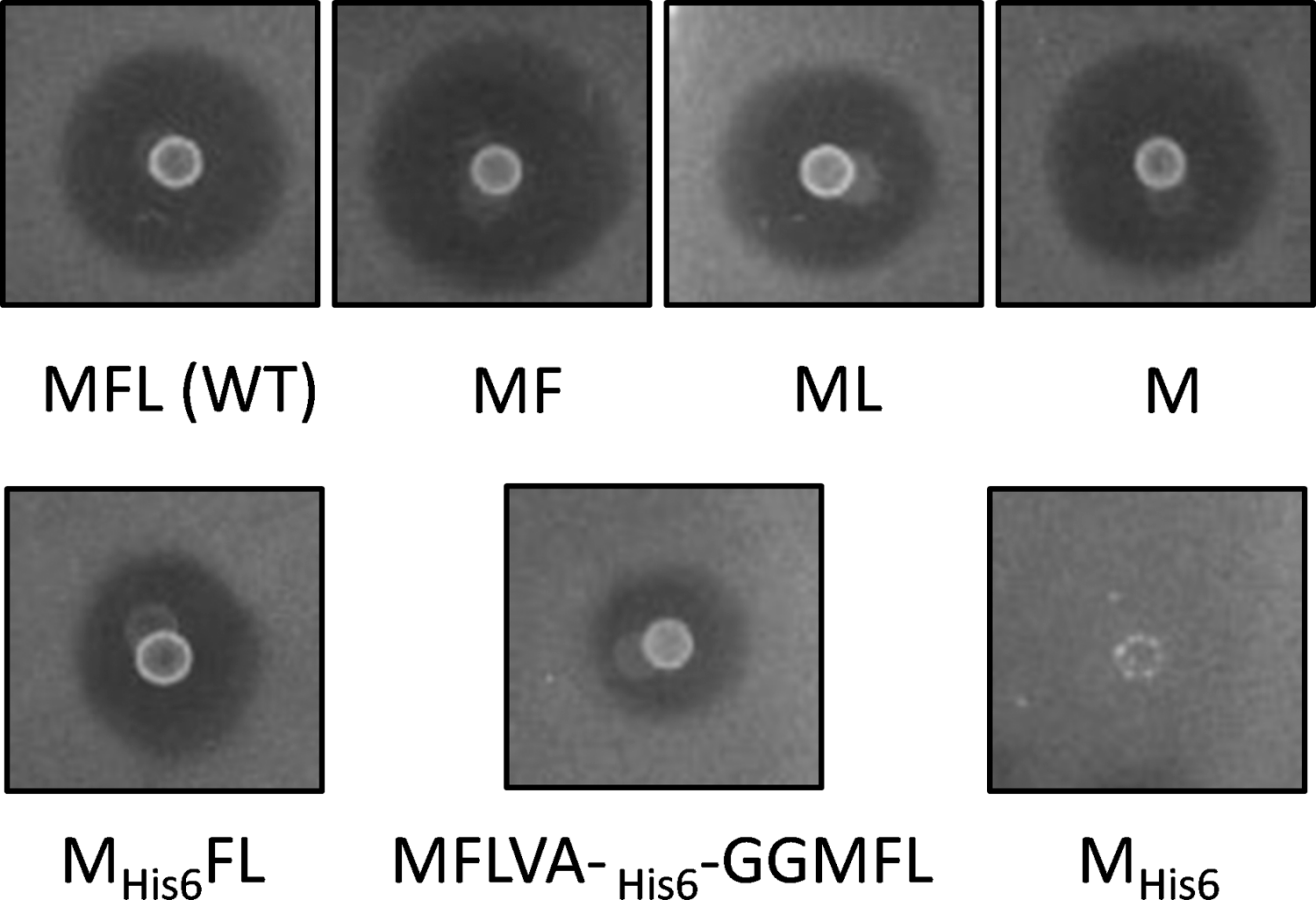
Antimicrobial activity of circularin A leader variants with leader truncations (or leader extensions). Indicator strain: *Lb. sake* ATCC 15521.

As shown in Figure 2, mutants with truncated leaders retained high antimicrobial activity, whereas mutants with extended leaders significantly reduced antimicrobial activity. Specifically, compared with the wild type, truncated leader MF slightly increased activity, a similar effect to the residue replacements MFA and MFW; leader ML showed a clearly decreased activity level; however, the leader of just one residue (Met) showed comparable activity levels to wild type. This is quite surprising since it has been reported that any leader truncations in Ent53B disabled antimicrobial activity.^27^ The leader peptide of Ent53B was considered to be essential for maintaining the overall conformation of the precursor and mutations in the leader probably resulted in the alteration of substrate conformation, thereby influencing its interaction with the biosynthetic enzyme(s). To our knowledge, we now demonstrate for the first time that a leader of only one residue (i.e., Met) is sufficient for circular bacteriocin biosynthesis, at least in the case of circularin A. It is still an open question whether leader processing between circular bacteriocins with long and short leaders shares a common feature. Mutagenesis studies of mersacidin, a class II lanthipeptide, suggested that the deletion of certain residue(s) in its leader mostly leads to complete loss of activity except mutant ΔG-6 that, however, also interfered with MrsT cleavage in (the first-step) leader processing.^39^

Notably, the leader-extended mutants with the addition of a 6xhis-tag (M_His6_ and M_His6_FL) significantly decreased activity compared with their respective leader variants (M and MFL). This may indicate that the addition of a 6xhis-tag to the leader sequence interfered with leader processing of the biosynthetic enzyme(s), thereby reducing the efficiency of bacteriocin production yields. The leader variant M_His6_ completely disabled bacteriocin production, which was similar to the effects of substitutions with charged residues at position F-2 (leader variants MKL and MDL). Interestingly, leader variant MFLVA-_His6_-GGMFL retained bacteriocin production (Figure S4), although its leader was extended greatly (16-aa leader compared with the original 3-aa leader). Future studies will be conducted with this mutant to investigate the level of peptide modification in the context of knockout of certain biosynthetic protein(s), since the his-tag facilitates either peptide purification for the precursor peptide in the absence of peptide modification, or for the leader peptide in the case of leader cleavage (and peptide maturation). Moreover, the leader variant MFLVA-_His6_-GGMFL has two potential leader cleavage sites, i.e., the wild-type leader sequence MFL. It is still a mystery how the biosynthetic enzyme(s) of circularin A recognizes the cleavage site, especially for leader mutants with altered lengths, such as leaders MF, ML, M, and MFLVA-_His6_-GGMFL. This gives us a strong indication that the recognition signal of the biosynthetic enzyme(s) probably lies in the core peptide rather than in the leader peptide. Thus, site-directed mutagenesis was performed on the N- and C-terminal residues, as well as their neighboring residues, of the circularin A core peptide.

### Site-Directed Mutagenesis of N- and C-Terminal and Their Neighboring Residues

**Figure 3:**
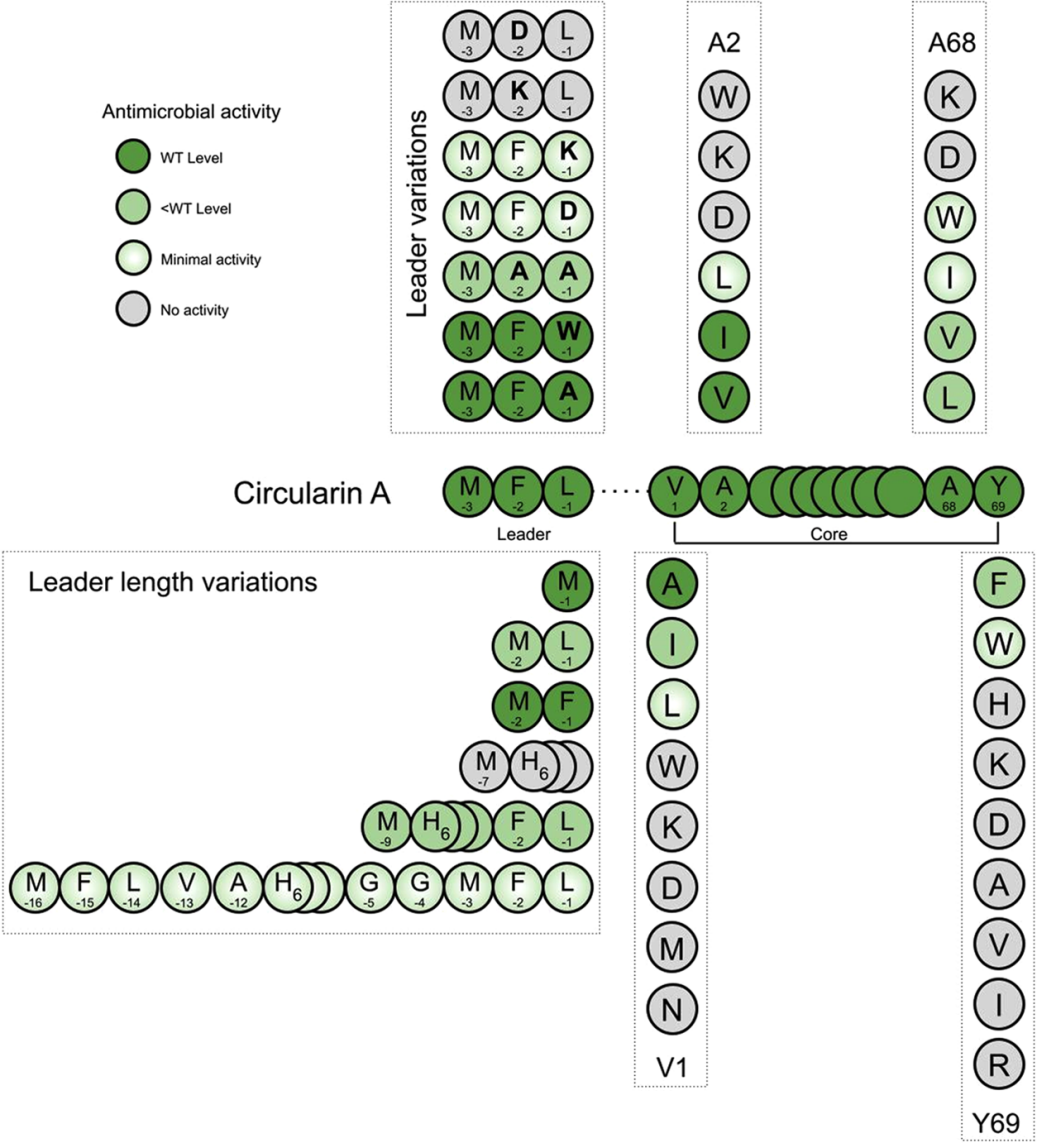
Overview of all of the mutants generated in this study and their effects on antimicrobial activity against *Lb. sake* ATCC 15521. The relative activity of each mutant was indicated based on the size of the inhibition zone in the activity assay.

Circular bacteriocins are characterized by head-to-tail ligation, and the ligation site is located within one helical structure. Circularin A is a subgroup I circular bacteriocin. Amino acid sequence alignment of subgroup I circular bacteriocins reveals that the N- and C-terminal residues are hydrophobic residues, often with a Leu residue at the N-terminus and a Trp residue at the C-terminus (Figure S1). In this study, four residues close to the ligation site were subjected to site-directed mutagenesis, which included the first two residues in the N-terminus of the core peptide and the last two in the C-terminus. According to the sequence alignment, the most abundant residues at these four positions are Leu, Ala, Ala, and Trp, accounting for 73, 47, 80, and 73%, respectively. In the case of circularin A, the residues at these four positions are Val1, Ala2, Ala68, and Tyr69. All of the mutant variants generated in this study and their effects on antimicrobial activity were overviewed in Figure 3.

#### Site-Directed Mutagenesis of N- and C-Terminal Residues Val1 and Tyr69

**Figure 4:**
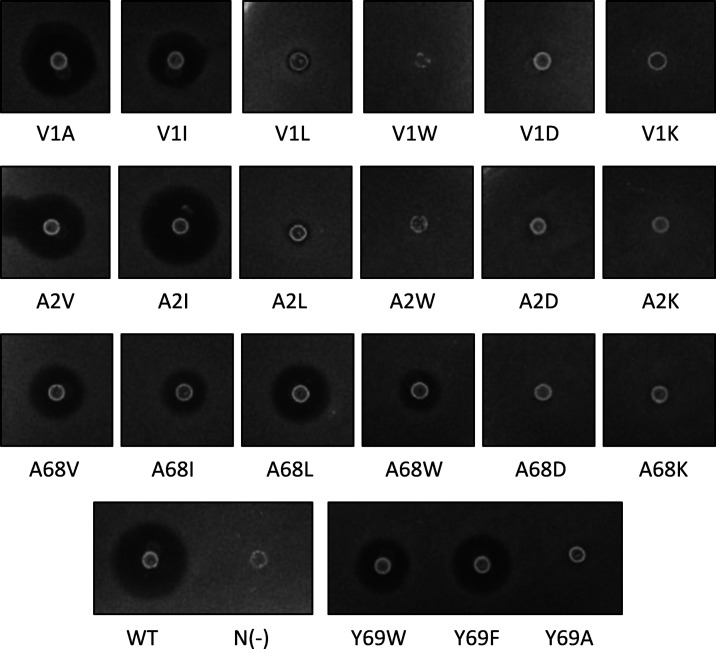
Variable effects of mutations at V1, A2, A68, and Y69 positions on circularin A biosynthesis and bioactivity. Indicator strain: *Lb. sake* ATCC 15521. Note: the activity levels shown here basically corresponded to their production levels (estimated by gel-based quantification), except that a large discrepancy was found in V1A that had a much lower yield relative to its activity level.

Based on residue properties, site-directed substitutions of residues Val1 and Tyr69 were performed with residues from various categories (Table 2). The effects of these mutants on antimicrobial activity are shown in Figure 4. For all tested Val1 and Tyr69 mutants, the resulting peptide derivatives retained activity only when the residues mutated to were similar to wild type, including the derivatives of Val1 mutated to the short-sidechain hydrophobic residues Ala and Ile (mutants V1A and V1I), as well as the derivatives of Tyr69 mutated to aromatic residues Phe and Trp (mutants Y69F and Y69W).

**Table 2:** Effect of Mutation at the Circularization Site (V1 and Y69) on the Production Yield of Mature Circularin A Derivative

	mutation properties/relative production yield (%)^a^												
	hydrophobic								hydrophilic				
	hydrophobic					aromatic			uncharged	charged			
position	A	V	I	L	M	F	Y	W	N	D	K	R	H
V1	+	++	+	+/–	–	ND	ND	–	–	–	–	ND	ND
Y69	–	–	–	ND	ND	++	++	+	ND	–	–	–	+/–

a Wild-type circularin A production was set as 100% and used for comparison. The relative peptide yield of each mutant was estimated based on the amount of peptide obtained from peptide purification, and was shown on a scale of 0–2% (−), 2–15% (+/−), 15–50% (+), and 50–100% (++). ND, mutation not done.

Specifically, of all the tested Val1 mutants, the highest activity was found in mutant V1A with an activity level comparable to wild type, followed by mutant V1I that retained less than half that of wild type (Figure 4). However, the activity of mutant V1L decreased significantly to almost invisible in the overlay activity assay, and no antimicrobial activity was observed for other Val1 mutants including V1M, V1W, V1N, V1K, and V1D (Figure S5A). Mutagenesis studies in Ent53B suggested that Leu1 mutations with short-sidechain hydrophobic residues (Ala, Ile, and Val) also produced active Ent53B derivatives, whereas Leu1 mutations with aromatic residues Phe and Tyr showed different effects: Leu1 mutation with helix-former Phe produced active Leu1Phe derivative of Ent53B and Leu1 mutation with helix-breaker Tyr blocked the derivative production; moreover, Leu1 mutations with the negatively charged residue (Glu) and helix-breaker residues (Gly and Pro) disabled Ent53B derivative production.^27^ The mutant M1A of AS-48 produced the mature derivative but with a strikingly low production.^35^

For peptide production yields, all of the Val1 variants had a production level less than half that of the wild type (Table 2). Leu1 variants of Ent53B also decreased the production yield significantly, especially for mutants L1V and L1A, which had a relative production of approximately 2 and 13%, respectively.^27^ Notably, mutant V1A of circularin A had a much lower level of bacteriocin yield but a comparable activity level to the wild type, suggesting that the alanine substitution at position Val1 reduced the production level of the mature bacteriocin that likely resulted from inefficient processing of this mutated precursor (this will be discussed further below); however, the V1A derivative of circularin A had an improved killing capability against the indicator strain *Lb. sake* ATCC 15521.

For the Tyr69 mutants, mutants of substitutions with aromatic residues (Phe and Trp) retained antimicrobial activity. However, Tyr69 mutations with short-sidechain hydrophobic residues (Ala, Val, and Ile) or charged residues (Lys, Arg, and Asp) abolished antimicrobial activity (Figure S5B). Further analysis suggested that these inactive Tyr69 mutations blocked the production of mature circularin A derivatives (Table 2). Despite being a basic residue at a low pH (pH < 6.5), histidine contains an aromatic ring in its sidechain, and thus mutant Y69H also showed a very low level of antimicrobial activity. Phenylalanine (Phe, F) is structurally closer to tyrosine (Tyr, Y) than any other residues including tryptophan (Trp, W), so mutant Y69F could be expected to have the best production yield and activity among all tested Tyr69 mutants. Mutation W70A of AS-48 has been the only mutant investigated at the C-terminal residues of other circular bacteriocins, and this mutation resulted in the production of three forms of the W70A derivative: circular, oxidized, and linear; the circular W70A derivative had comparable activity levels to wild-type AS-48, and the oxidized form had a partial loss of activity.^35^ Based on our observations of the chromatography data, we found that in addition to the circular form of circularin A, the oxidized forms were also commonly seen (Figure S6), as in the case of AS-48. However, we did not see any linear forms of circularin A in our sample analysis.

#### Site-Directed Mutagenesis of Ala2 and Ala68 Residues

**Figure 5:**
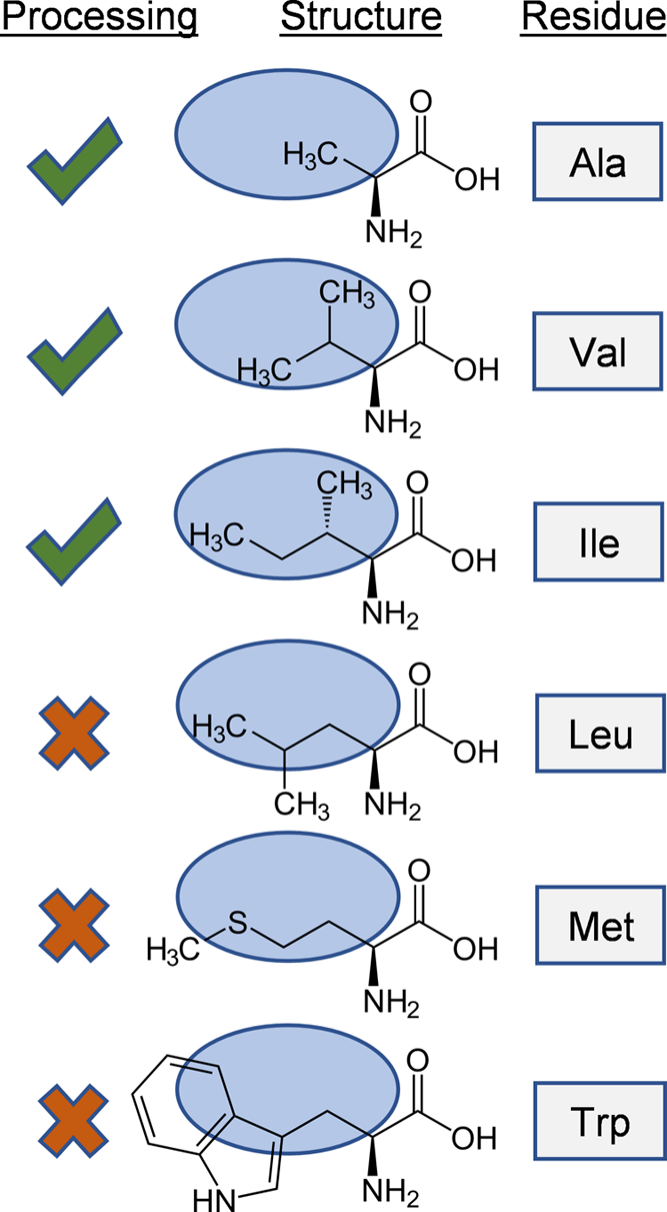
Proposed mechanism of steric hindrance in the biosynthetic processing of Ala2 (and Val1) derivatives of circularin A. In this theory, the binding pocket size (indicated by the blue region) of the biosynthetic enzyme is critical: residues with bulky sidechains at this position inhibit bacteriocin maturation. The later branched sidechain of Leu, compared with Ile, also severely affects production yield.

Site-directed substitutions of Ala2 and Ala68 residues were performed with either hydrophobic residues or hydrophilic charged residues (Table 3). The effects of these mutants on antimicrobial activity were investigated (Figure 4). The results revealed that antimicrobial activity was largely retained for Ala2 substitutions with short-sidechain hydrophobic residues Val and Ile (mutants A2V and A2I), and mutant A2I even had slightly larger inhibition zone than the wild type. However, mutant A2L almost completely lost activity although Leu is the same size as Ile. Protein gel analysis (Figure S7) revealed that the peptide yield of the A2I derivative increased compared with that of the wild type, while the peptide yield of the A2L derivative was severely disturbed, and thus the activity loss of mutant A2L was caused by the low production yield of the mature A2L derivative. This observation indicates that the later Cγ branching of a Leu residue (opposed to Cβ branching in Val and Ile) at position 2 may interfere with the function of the biosynthetic enzyme(s) in peptide processing through steric hindrance effects (Figure 5), as is likely the case with the V1L mutant. Moreover, substitutions of Ala2 with the aromatic residue Trp (W) or charged residues Asp (D) and Lys (K) disabled antimicrobial activity. For the Ala68 mutants, substitutions with the hydrophobic residues Val, Ile, Leu, and Trp (mutants A68V, A68I, A68L, and A68W) all retained antimicrobial activity, but their activity was significantly reduced to less than half that of the wild type (Figure 4). Gel analysis suggested that production yields of the A68 mutants were well consistent with their activity levels (Table 3). As in the case of Val1, Tyr69, and Ala2, replacement of Ala68 with the charged residues Asp (D) and Lys (K) also resulted in a complete loss of antimicrobial activity.

**Table 3:** Effect of Mutation at Positions A2 and A68 on the Production Yield of Mature Circularin A Derivative

	mutation properties/relative production yield (%)^a^						
	hydrophobic					hydrophilic, charged	
position	A	V	I	L	W	K	D
A2	WT	+++	+++	+/–	–	–	–
A68	WT	+	+/–	+	+/−	–	–

a Wild-type circularin A production was set as 100% and used for comparison. The relative peptide yield for each mutant was estimated based on the amount of peptide obtained from peptide purification and was shown on a scale of 0–2% (−), 2–15% (+/−), 15–50% (+), 50–100% (++), and >100% (+++).

The residues at positions Val1, Ala2, and Ala68 of circularin A are all hydrophobic residues with short sidechains. We found that antimicrobial activity was retained for substitutions of these residues with other short-sidechain hydrophobic residues such as Ala, Val, Ile, and Leu (Figure 4). However, the retained antimicrobial activity varied significantly among these mutants, especially for substitutions at these three positions with Ile and Leu, respectively. For the N-terminal residues Val1 and Ala2, their replacements with Leu lead to nearly complete loss of activity, while their substitutions with Ile largely retained antimicrobial activity. This indicated that these two residues are of crucial importance in interacting with (or binding to) the biosynthetic enzyme(s) of circularin A. For residue Ala68 at the C-terminus, its substitution with Ile significantly reduced antimicrobial activity, while mutant A68L preserved much better activity than A68I (Figure 4). Moreover, Ala68 substitution with the largest sidechain residue Trp retained activity, unlike tryptophan substitutions at Val1 and Ala2 positions. It seems very likely that the antimicrobial activity of Ala68 mutants could be retained as long as Ala68 is substituted with a hydrophobic residue.

In short, site-directed mutagenesis of terminal residues (Val1, Ala2, Ala68, and Tyr69) suggested that peptide production was preserved only for substituted mutants at these four positions with hydrophobic residues, which included mutants with helix-forming short-chain hydrophobic residues at positions Val1 and Ala2 of the N-terminus, aromatic residues at the C-terminal Tyr69 position, and the merely hydrophobic residues at the Ala68 position.

#### Mutations in Circularin A Can Lead to Degradation of the Precursor Peptide

**Figure 6:**
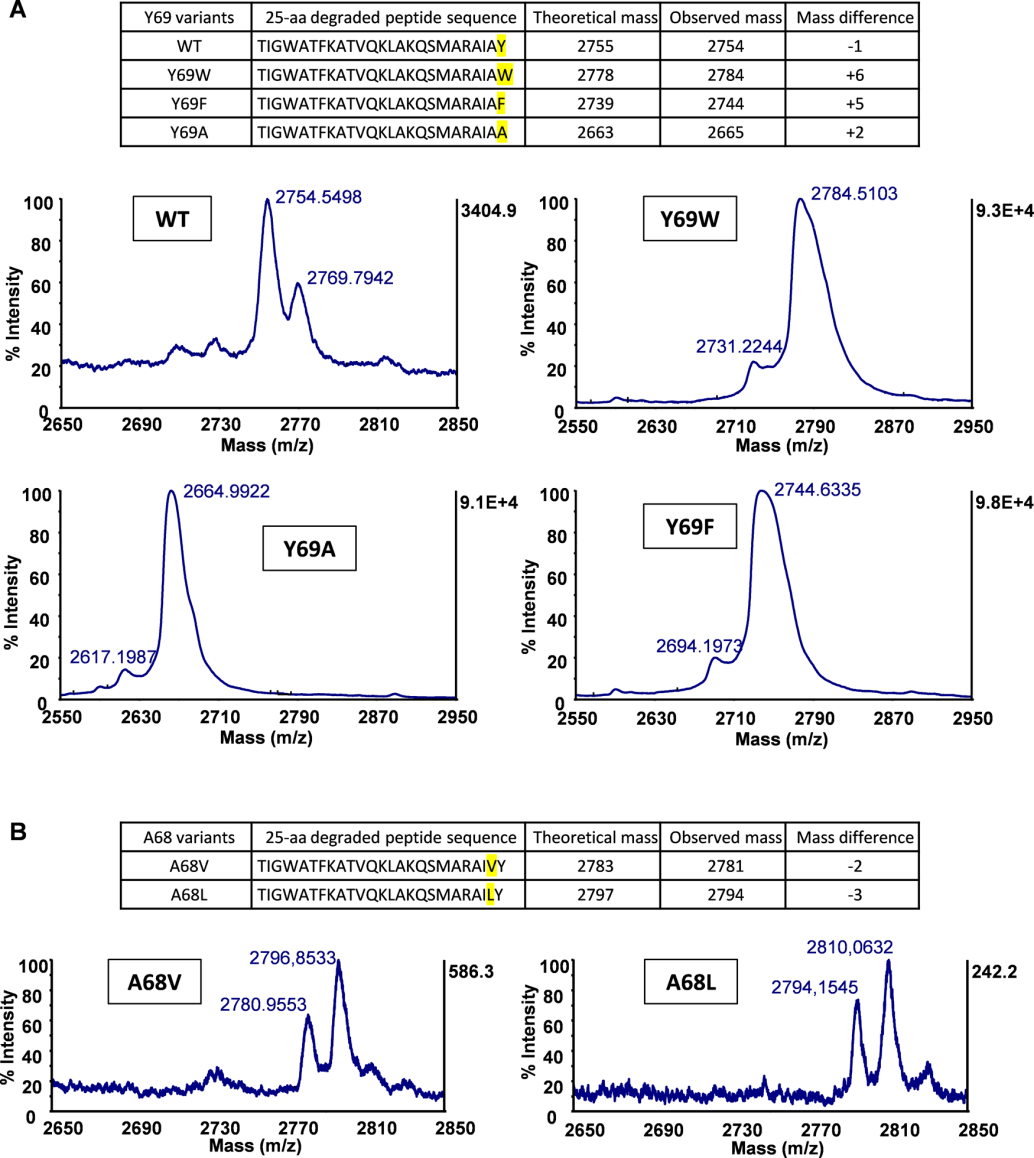
MOLDI-TOF spectra of the degraded fragments in various C-terminal Tyr69 (or Ala68) mutants. Oxidation was clearly seen in Ala68 variants (and perhaps other mutants as well). This is likely due to the oxidation of the Met residue in the degraded 25-aa peptide fragment.

As stated above, mutations of the circularin A core peptide (N- and C-terminal residues) generally decreased production yield and the resulting peptide derivatives retained activity only when mutated to residues similar to wild type. This is likely due to changes of sidechains in these substitutions that lead to changes of the substrate conformation, thereby interfering with the ability of the precursor peptide to interact with the substrate-binding cleft of the biosynthetic enzyme(s). Mutation generally slows down the modification rate of the biosynthetic enzyme(s) and results in inefficient modification of the precursor peptide (if the peptide can still be processed). Consequently, the precursor peptide might reside longer in the cell and easily get degraded, resulting in a lower bacteriocin yield. One piece of evidence for this speculation is that the (partial-)degradation of circularin A was observed in various mutants, especially in Val1 and Ala2 mutants. For example, gel analysis of the Ala2 mutants (Figure S8) revealed an intense peptide band corresponding to a peptide that was smaller and less hydrophobic than the target bacteriocin, circularin A. Subsequent MALDI-TOF analysis suggested that this peptide fragment corresponded to the size of the 25-aa C-terminal fragment of circularin A (amino acid sequence: TIGWATFKATVQKLAKQSMARAIAY), which was also endorsed by the variable sizes of degraded fragments in C-terminal Tyr69 (or A68) mutants (Figure 6). These observations support our hypothesis that the intrinsic structure of the precursor, rather than the leader recognition, is the key to circularin A maturation, and any disruptions of the innate peptide structural propensity would lead to inefficient modification and (partial or complete) degradation of the precursor peptide (Figure S9).

## Conclusions

In this study, systematic site-directed mutagenesis was performed at positions near the leader cleavage site and the circularization ligation site. Residue substitution assays of the leader sequence suggested that hydrophobic residues are more favored than charged residues in the leader of circularin A for bacteriocin biosynthetic processing and that the aromatic residue F-2 is more important than residue L-1 in retaining efficient bacteriocin production. Leader truncation/extension assays revealed that the leader length of circularin A was quite flexible in retaining bacteriocin production: peptide production was achieved by mutants with a leader of just one residue (M) or a 16-aa leader (MFLVA-_His6_-GGMFL). The His-tag in the leader (potentially) enables future investigations into the mechanism of action of the modification machinery. Residue substitutions of the terminal residues (core peptide) suggested that peptide production was retained only for substituted mutants with residues similar to that of the wild type, especially for substitutions of Val1, Ala2, and Tyr69. This indicates that these three residues are probably involved in the binding of the biosynthetic enzyme(s). Modeling efforts and protein engineering of its biosynthetic proteins are now required to investigate this further. Overall, we extensively investigated the mutational effects of the leader and the N- and C-terminal residues on circularin A production. Our findings provide new insights into the biosynthesis of circular bacteriocins and will facilitate further experimental designs to elucidate the biosynthetic mechanism of circular bacteriocins. This ultimately brings us closer to designed circular peptides with desired properties produced by fermentation.

## Methods

### Bacterial Strains and Growth Conditions

*L. lactis* NZ9000 was used as the host strain for molecular cloning and bacteriocin expression. It was grown at 30 °C in M17 broth supplemented with 0.5% (w/v) glucose (GM17). For cell growth on plates, 1.5% agar was added to the medium. When required, chloramphenicol and erythromycin were used at 5 μg/mL each for *L. lactis* NZ9000. *Lb. sake* ATCC 15521 was used as the indicator strain in the activity assay. It was grown at 30 °C in De Man Rogosa and Sharpe broth (MRS). For bacterial growth on plates in activity assays, *Lb. sake* ATCC 15521 was grown at 30 °C in MRS broth supplemented with 0.8% agar. All of the media and chemicals were purchased from Sigma-Aldrich unless stated otherwise.

### Molecular Cloning and Site-Directed Mutagenesis in *cirA*

The techniques of standard molecular cloning were performed as previously reported.^40^ Molecular cloning and site-directed mutagenesis were designed and performed based on the previous reported two-plasmid production system (pNZ-*cirA* & pTLR4-*cirBCDE*).^34^ Briefly, plasmid pNZ-*cirA* was used as the template to introduce desired mutations, and primers were designed by applying the inverse PCR technique. All primers used in this study were ordered from Biolegio (Nijmegen, the Netherlands) (Table S2). PCR fragments were isolated using the NucleoSpin gel & PCR cleanup kit (Bioke, Leiden, the Netherlands). The purified PCR fragments were ligated using either Gibson Assembly Master Mix or USER Enzyme (Bioke, Leiden, the Netherlands) according to the manufacturers’ instructions, or T4 ligase at 22 °C for 3–5 h. Each of the constructed *cirA* mutants was first transformed into “empty” *L. lactis* NZ9000 by electroporation with a Gene-Pulser (Bio-Rad) as described earlier.^41^ A selection of the resulting colonies was inoculated in liquid GM17 medium with an appropriate antibiotic (5 μg/mL chloramphenicol in this case) and grown overnight at 30 °C. The fresh overnight cell culture was then subjected to plasmid isolation (NucleoSpin Plasmid EasyPure kit). The successful insertion of the desired mutation was confirmed by gene sequencing (Macrogen Europe). Eventually, the correctly sequenced *cirA* mutant was transformed into *L. lactis* NZ9000 already harboring the biosynthetic proteins (CirBCDE). Glycerol (20%, v/v) stocks of the constructed strains were stored at −80 °C.

### Bacteriocin Production and Antimicrobial Activity Tests

For bacteriocin production, the expression conditions of *cirA* mutants were slightly modified from the previous reported method,^34^ and two times of nisin induction was applied during the expression period. Specifically, *L. lactis* hosting the bacteriocin variant was first inoculated in normal GM17 medium and grown overnight at 30 °C. The next day, 5 mL of fresh overnight culture was centrifuged at 6000*g* for 3 min. After that, the cells were resuspended in minimum essential medium^42^ (MEM) and subjected to a second centrifugation to remove any residual GM17 medium. Then, the cells were transferred into 100 mL of MEM supplemented with 2.2% glucose and 5 ng/mL nisin and grown at 30 °C for ∼4 h (OD600: 0.3–0.5). When the cells reached the early exponential growth stage, 5 ng/mL nisin was added again to the culture. Then, the cells were induced at 30 °C for another 16–20 h. Finally, the cell culture was centrifuged at 8000*g* for 15 min and the supernatant was collected for subsequent C18 open-column purification. The C18 purification was performed as previously reported.^34^

Antimicrobial activity of circularin A variants was assessed by the colony overlay assay as reported previously.^34^ Briefly, the bacteriocin host strain (*L. lactis*) was first spotted on GSM17 agar plate that contained 0.5% glucose, 0.5 M sucrose, 1.5% (w/v) agar, as well as 5 ng/mL nisin for peptide induction. The plate was then incubated at 30 °C for 16–20 h to allow the host strain grow and produce the peptide, and this plate served as the first layer in the colony overlay assay. For the second layer, the indicator strain *Lb. sake* ATCC 15521 was first grown in MRS liquid medium at 30 °C for 36–48 h, then the obtained cell culture (OD600: 0.8–1.2) was diluted 1000-fold into MRS medium supplemented with 0.8% agar. This second-layer MRS medium seeded with the indicator strain was poured on top of the first-layer GSM17 agar plate where the producer strain was grown. Ultimately, the two-layer testing plate was incubated at 30 °C for 36–48 h to allow the growth of the indicator strain.

### Tricine-SDS-PAGE

The production levels of circularin A variants were assessed by tricine-SDS-PAGE. The gels were prepared as previously reported.^43^ Briefly, tricine-SDS-PAGE analysis was performed with a 16% separating gel and a 4% stacking gel. The amount of peptide used for the gel analysis was purified from 20 mL of cell culture. The eluted peptide sample from C18 purification was freeze-dried and dissolved in 20 μL of milli-Q water (1000-fold concentrated). A 5 μL loading buffer (10% SDS, 0.5% bromophenol blue, 50% glycerol, 250 mM Tris-HCl, pH 6.8) was added into the peptide sample, and the mixture was treated at 50 °C for 30 min. A 5 μL Unstained Low Range Protein Ladder (PageRuler, Thermo Fisher) was used as a protein marker. After gel separation, the staining and destaining procedures were performed as previously reported.^34^ Peptide bands were viewed in Image Lab 3.0 and peptide yields were estimated by band intensities.

### MALDI-TOF and Liquid Chromatography–Mass Spectrometry (LC–MS) Analyses

The peptide samples obtained from C18 purification were first freeze-dried and dissolved in a small volume (e.g., 50 μL for peptide purified from 50 mL of cell culture) of 20% acetonitrile solution to concentrate 1000-fold. The concentrated samples were analyzed by matrix-assisted laser desorption/ionization-time-of-flight (MALDI-TOF) or liquid chromatography–mass spectrometry (LC–MS). Briefly, 1 μL of the sample was spotted on the MALDI-TOF target and dried at room temperature. Subsequently, 1 μL of the matrix solution of 5 mg/mL α-cyano-4-hydroxycinnamic acid (or 10 mg/mL sinapic acid) was spotted on the top of the peptide sample. After the sample was dried, MALDI-TOF MS was performed using a 4800 Plus MALDI-TOF/TOF analyzer (Applied Biosystems) operating in the linear-positive mode. The retrieved mass spectra were visualized in Data Explorer 4.9.

LC–MS analysis was performed by the Waters ACQUITY UPLC H-Class PLUS Bio System connected with a Waters Xevo G2 Q-Tof (Quadrupole time-of-flight). The analytical column was an ACQUITY UPLC Protein BEH C4 Column (150 mm by 2.1 mm, 1.7 μm particle size, 300 Å pore size). First, the freeze-dried sample was concentrated 1000-fold in 20% acetonitrile solution supplemented with 0.1% formic acid (FA). The insoluble material was removed by centrifugation at 12,000*g* for 20 min. A 6 μL sample was carefully taken from the supernatant and subjected to the LC–MS system. Acetonitrile was used for peptide elution. The mobile phase consisted of 0.1% FA in water (solution A) and acetonitrile (solution B). All of the solutions were of ULC/MS-grade quality (Biosolve). The procedure of each sample run was as follows: 0–2 min, 5% solution B; 2–10 min, 5–95% solution B (solution B increased from 5 to 95% at a linear rate); 10–12 min, 95% solution B; 12–12.1 min, 95–10% solution B (solution B decreased from 95 to 10% at a linear rate); and 12.1–19 min, 10–5% solution B. The flow rate was 0.3 mL/min. The mass detector operated in the positive ionization mode, and the scanning range of Q-Tof MS was *m*/*z* 200–7000. The retrieved spectrum was viewed and analyzed in MassLynx 4.1.

### Sequence Alignment Analysis

The amino acid sequence of circularin A was aligned with sequences of other characterized subgroup I circular bacteriocins. The candidate sequences are listed in Table S3. The sequence alignment was performed using Clustal Omega (https://www.ebi.ac.uk/Tools/msa/clustalo/). The alignment result was viewed and edited with Jalview^44^ (version 2.11.2.1).

## Supporting Information Available

Amino acid sequence alignment of the characterized subgroup I circular bacteriocins (S1); MALDI-TOF spectra of circularin A leader variants with site-directed substitutions (S2); tricine-SDS-PAGE to determine the production levels of circularin A in various leader mutants (S3); MALDI-TOF spectrum of peptide purified from the 16-aa leader variant (MFLVA-_His6_-GGMFL) (S4); antimicrobial activity of Val1 (or Tyr69) mutants with site-directed substitutions in circularin A (S5); detected masses from the LC–MS analysis of the wild-type circularin A (S6); tricine-SDS-PAGE to determine the production levels of mature circularin A derivatives in Ala2 mutants (S7); peptide degradation of various A2 mutants (S8); proposed scheme of possible biosynthetic processing of circularin A derivatives (S9); short-leader circular bacteriocins and their leader sequences (S10); oligonucleotides used in this study (S11); and amino acid sequences of the characterized subgroup I circular bacteriocins (S12) (PDF)
