# Supplementary material for: Leader- and Terminal Residue Requirements for Circularin A Biosynthesis Probed by Systematic Mutational Analyses
Source: ACS Synth Biol. 2023 Mar 1;12(3):852–62. doi: 10.1021/acssynbio.2c00661 (PMC10028692; doi:10.1021/acssynbio.2c00661)
Supplement: Supplementary file 1 — sb2c00661_si_001.pdf [file sb2c00661_si_001.pdf]

Supporting Information

Leader- and terminal residue requirements for circularin A biosynthesis probed by systematic mutational analyses

Fangfang Liu, Auke J. van Heel and Oscar P. Kuipers*

Department of Molecular Genetics, Groningen Biomolecular Sciences and Biotechnology Institute, University of Groningen, 9747 AG Groningen, the Netherlands

*** Correspondence:**
Oscar P. Kuipers
o.p.kuipers@rug.nl

S1. Amino acid sequence alignment of the characterized subgroup I circular bacteriocins.

The alignment was performed using Clustal Omega with default settings. The color coding was shown in Jalview by setting conservation with percentage identity above 30%.

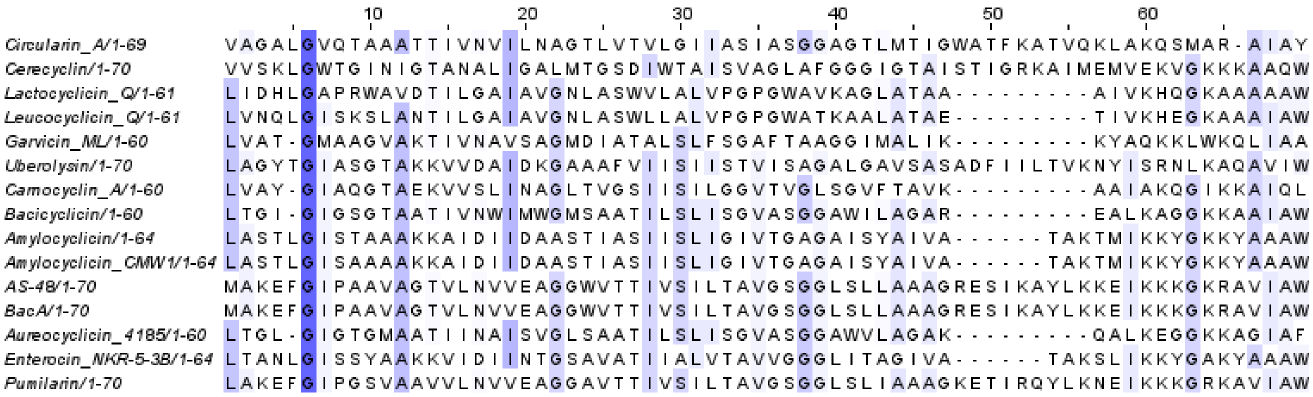

S2. MALDI-TOF spectra of circularin A leader variants with site-directed substitutions.

All these active leader variants produce the fully modified circularin A: the leader cleavage site of these leader variants is always in front of Val1, and the mass difference of a few Daltons is likely because the sample contains a mixture of WT bacteriocin and bacteriocin with 1x (or 2x) oxidation of the Met residue(s).

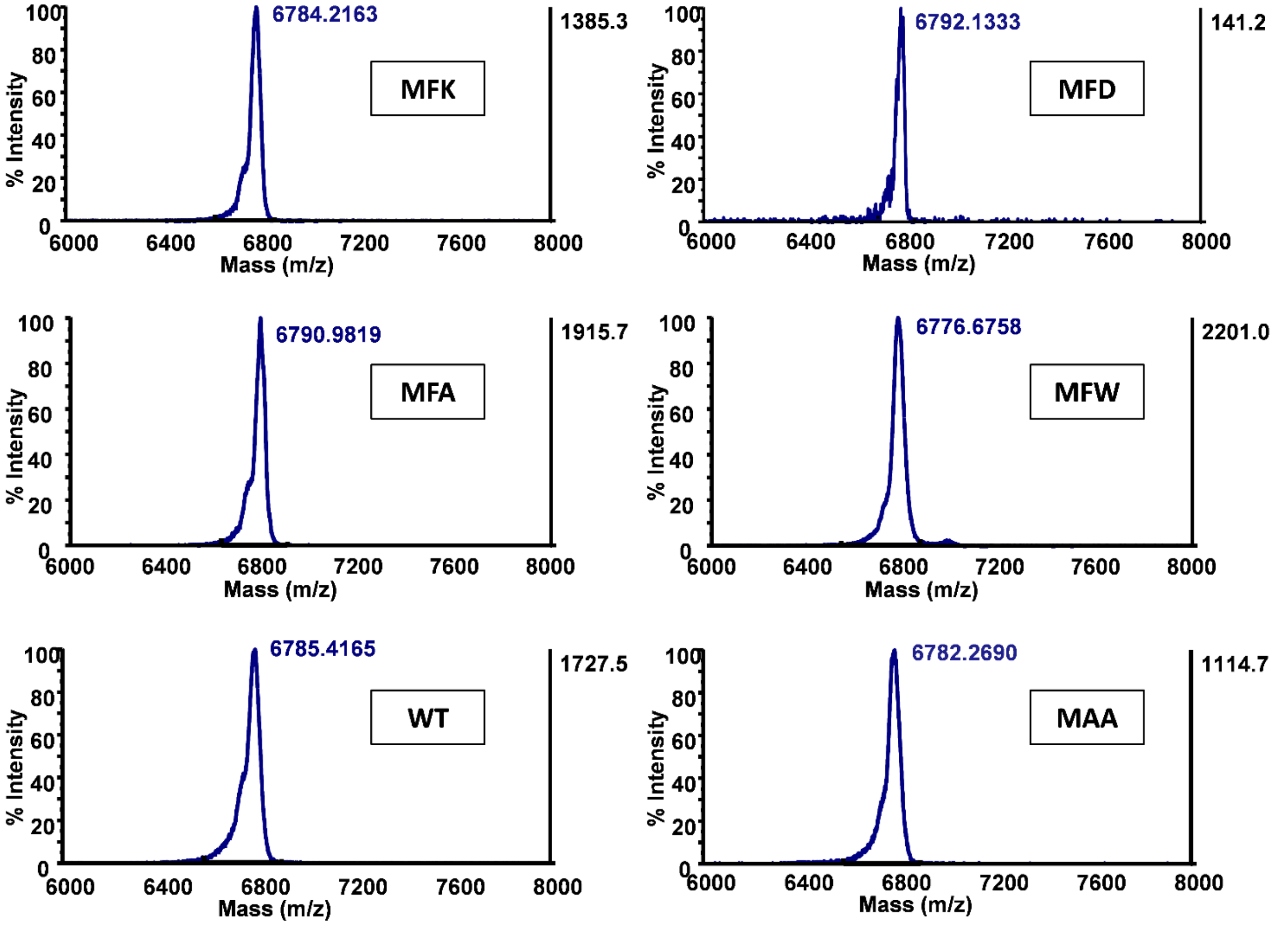

S3. Tricine-SDS-PAGE to determine the production levels of circularin A in various leader mutants.

The wild-type peptide showed a peptide band corresponding to a size close to 5K Dalton (Da). MFK seemed to have better yield relative to its activity level. However, the sample of MFK was also more prone to degradation (shorter peptides that run below the mature peptide were observed), which might explain the lower activity detected for MFK.

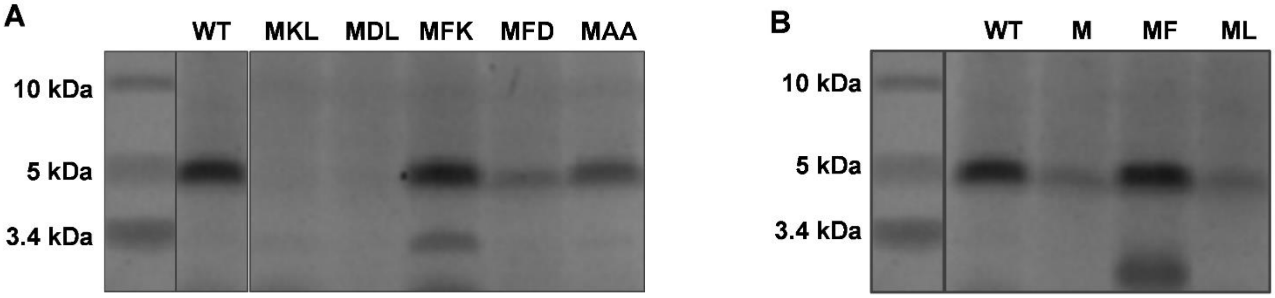

S4. MALDI-TOF spectrum of peptide purified from the 16-aa leader variant (MFLVA- $_{\text{His6}}$-GGMFL).

The theoretical mass of the mature circularized circularin A (WT) is 6771.05 Da. The mass difference between peptides produced from this 16-aa leader variant and the 3-aa leader WT is less than 7 Dalton, suggesting the correctly circularized bacteriocin production for this leader variant.

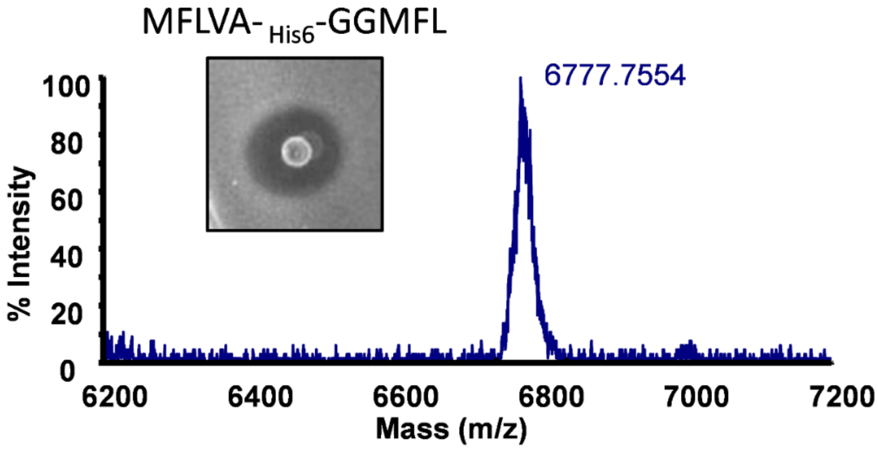

S5. Antimicrobial activity of Val1 (or Tyr69) mutants with site-directed substitutions in circularin A. The indicator strain: *Lactobacillus sake* ATCC 15521.

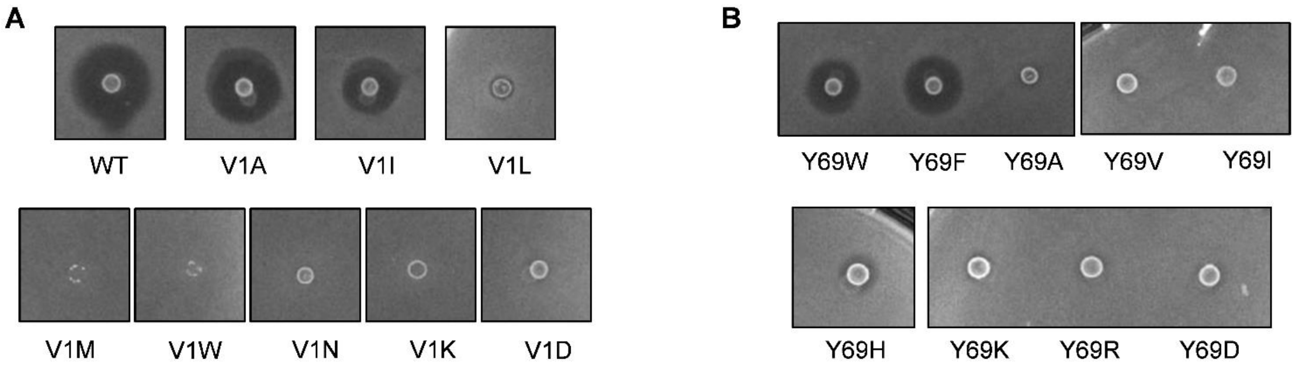

S6. Detected masses from the LC-MS analysis of the wild-type circularin A.

Three forms of fully modified circularin A were identified: the circular mature bacteriocin (WT), WT with 1 oxidation and WT with 2 oxidations.

Variant form	Mw	M/Z		
		$+3H^+$	$+4H^+$	$+5H^+$
**Circular/** mature	WT 6771	2258	1693,75	1355,2
**Circular/** 1 oxidation	WT+16 6787	2263,3	1697,75	1358,4
**Circular/** 2 oxidations	WT+32 6803	2268,67	1701,75	1361,6
**Linear** No leader	WT+18 6789	2264	1698,25	1358,8
**Linear** With leader	7180.58	2394,52	1796,14	1437,11

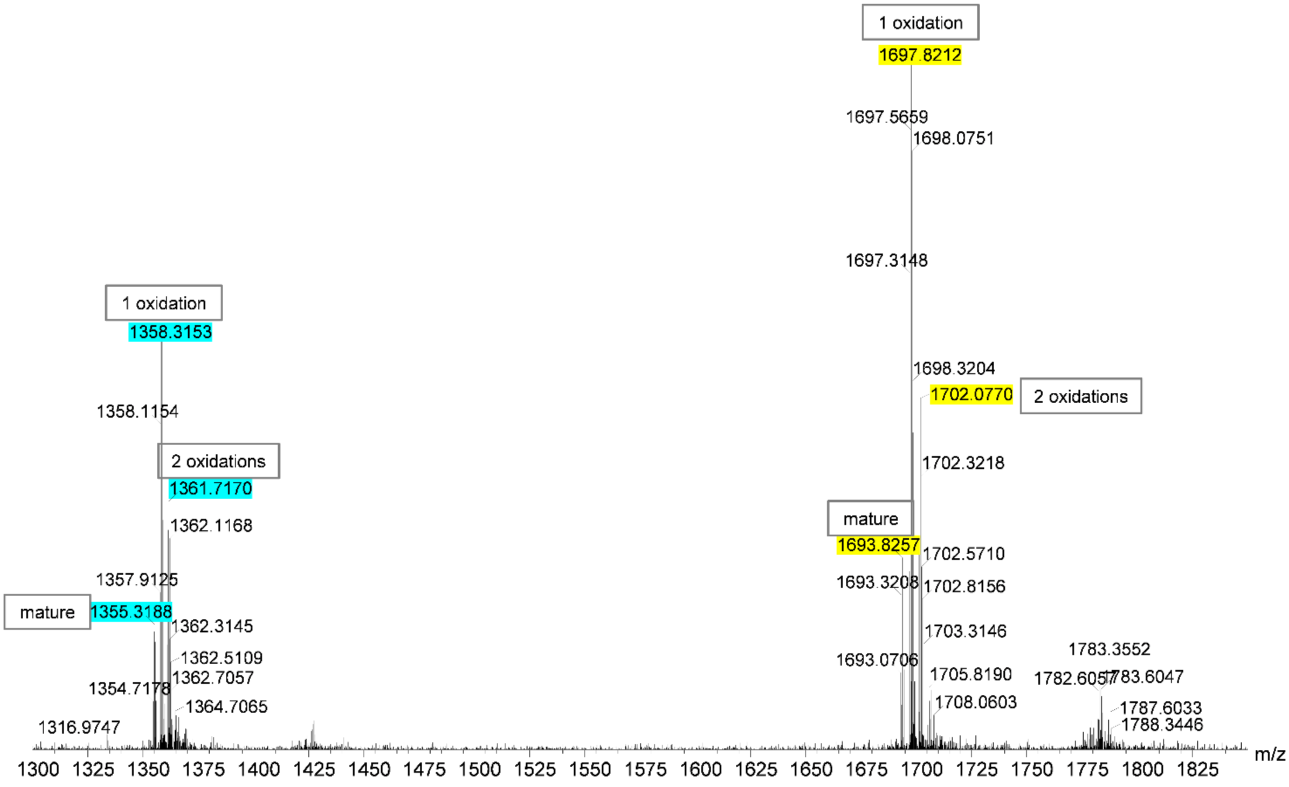

S7. Tricine-SDS-PAGE to determine the production levels of mature circularin A derivatives in Ala2 mutants.

The wild type showed a single band of the target peptide (corresponding to a size close to 5K Dalton, the second lowest band of the protein ladder).

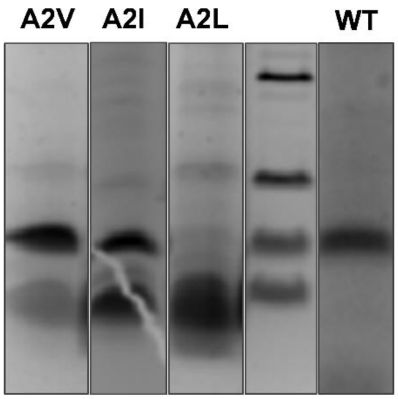

S8. Peptide degradation of various Ala2 mutants

These peptide fragments were eluted in 50% solvent fraction from C18 purification and they ran slightly below 5K Dalton (Da). The mature circularin A was often eluted in 80% solvent fraction and showed a peptide band corresponding to a size close to 5K Dalton (Da). Compared to Ala2 mutants, the wild type (WT) was less degraded.

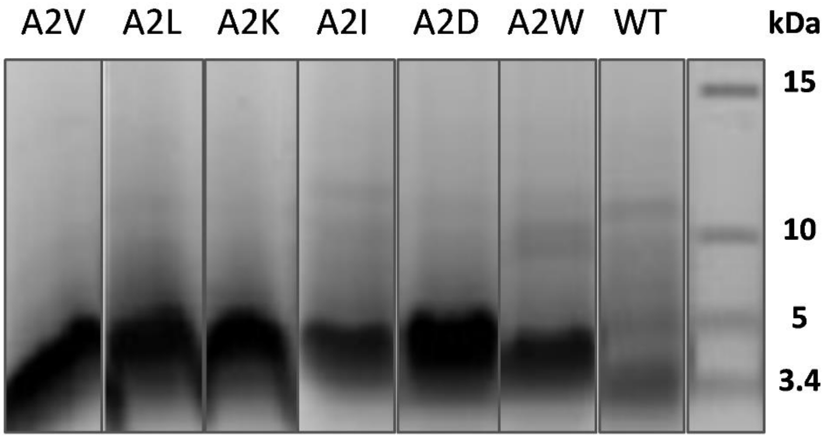

S9. Proposed scheme of possible biosynthetic processing of circularin A derivatives.

In this theory, the maintaining of the intrinsic structure of the precursor in mutants is critical for biosynthetic processing of the derivatives with the cognate enzyme(s).

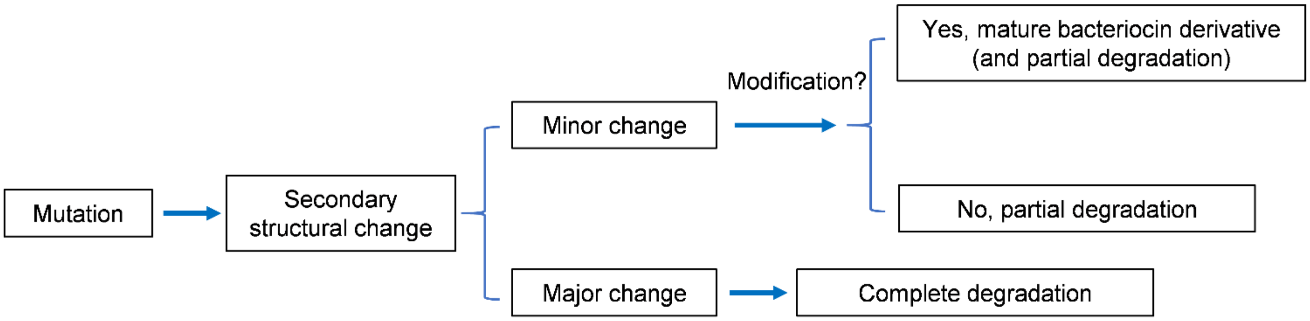

S10 (Table S1). Short-leader circular bacteriocins and their leader sequences

**Circular bacteriocin**	**Leader sequence [a]**					
	-6	-5	-4	-3	-2	-1
Circularin_A				M	F	L
Cerecyclin			M	L	F	N
Uberolysin	M	D	I	L	L	E
Lactocyclicin_Q					M	K
Leucocyclicin_Q					M	F
Garvicin_ML				M	F	D
Carnocyclin_A			M	L	Y	E
Bacicyclicin XIN-1			M	L	F	E
Aureocyclicin 4185			M	L	L	E

[a] aromatic residues are highlighted in yellow and charged residues in green.

S11 (Table S2). Oligonucleotides used in this study

Variant	Primer	Sequence (5'→3')
Leader variant		
M	P01	ACTCACCAUGGTTGCAGGAGCACTAGG
	P02	ATGGTGAGUGCCTCCTT
ML	P03	ACTCACCAUGTTAGTTGCAGGAGCACTAGGC
	P04	ATGGTGAGUGCCTCCTT
MF	P05	ACTCACCAUGTTTGTTGCAGGAGCACTAGGC
	P04	ATGGTGAGUGCCTCCTT
M_{His6}	P06	ACCATCACCAUCATGTTGCAGGAGCACTAGG
	P07	ATGGTGATGGUGATGCATGGTGAGTGCCTCCTT
M_{His6}MFL	P08	ACCATCACCAUCATATGTTTTTAGTTGCAGGAGCAC
	P09	ATGGTGATGGUGATGCATGGTGAGTGCCTCCTT
MFLVA-$_{His6}$-GGMFL	P10	CATCATCACCACGGTGGTATGTTTTTAGTTGCAGGAGCACTAG
	P11	GTGATGAGCAACAAGGAACATTTTGAGTGCCTCCTTATAATTTATTTTGTAGTTCC
MDL	P12	AGGAGCACUAGGCGTGCAA
	P13	AGTGCTCCUGCAACTAAATCCATAATTAATCACC
MFD	P12	AGGAGCACUAGGCGTGCAA
	P14	AGTGCTCCUGCAACATCAAACATAATTAATCACC
MKL	P12	AGGAGCACUAGGCGTGCAA
	P15	AGTGCTCCUGCAACTAATTTCATAATTAATCACC
MFK	P12	AGGAGCACUAGGCGTGCAA
	P16	AGTGCTCCUGCAACTTTAAACATAATTAATCACC
MAA	P12	AGGAGCACUAGGCGTGCAA
	P17	AGTGCTCCUGCAACTGCAGCCATAATTAATCACC
MFA	P45	GGAGCACTAGGCGTGCAA
	P50	TGCAACAGCAAACATGGTGAGTGCCTCCTT
MFW	P45	GGAGCACTAGGCGTGCAA
	P51	TGCAACCCAAAACATGGTGAGTGCCTCCTT
Bacteriocin derivative		
V1I	P18	TAAAAACATGGTGAGTGCCTCC
	P19	ATTGCAGGAGCACTAGGCG
V1L	P18	TAAAAACATGGTGAGTGCCTCC
	P20	TTAGCAGGAGCACTAGGCG
V1M	P18	TAAAAACATGGTGAGTGCCTCC
	P21	ATGGCAGGAGCACTAGGCG
V1N	P18	TAAAAACATGGTGAGTGCCTCC
	P22	AATGCAGGAGCACTAGGCG
A2K	P23	GTTTTTAGTTAAAGGAGCACTAGGCGTGCAA
	P24	CACGCCTAGTGCTCCTTTAACTAAAAACATGG
A2V	P25	GTTTTTAGTTGTTGGAGCACTAGGCGTGCA
	P26	ACGCCTAGTGCTCCTAAAACTAAAAACATGG
A68L	P27	TATGGCAAGAGCTATATTATACTAAGCTTTCTTTGAACCAA
	P28	GTTCAAAGAAAGCTTAGTATAATATAGCTCTTGCCATACTT

A68V	P29	TATGGCAAGAGCTATAGTTTACTAAGCTTTCTTTGAACCAA
	P30	GTTCAAAGAAAGCTTAGTAAACTATAGCTCTTGCCATACTT
A68K	P31	AAGTATGGCAAGAGCTATAAAATACTAAGCTTTCTTTGAACC
	P32	TTCAAAGAAAGCTTAGTATTTTATAGCTCTTGCCATACTTTGC
A68W	P33	GTATGGCAAGAGCTATATGGTACTAAGCTTTCTTTGAACC
	P34	GTACCATATAGCTCTTGCCATACTTTGCTTAG
Y69H	P35	TAAGCTTTCTTTGAACCAAAATTAGAAAAC
	P36	TTTGGTTCAAAGAAAGCTTAATGAGCTATAGCTCTTG
Y69I	P35	TAAGCTTTCTTTGAACCAAAATTAGAAAAC
	P37	TTTGGTTCAAAGAAAGCTTATATAGCTATAGCTCTTGCC
Y69R	P35	TAAGCTTTCTTTGAACCAAAATTAGAAAAC
	P38	TTTGGTTCAAAGAAAGCTTATCTAGCTATAGCTCTTGCC
V1A	P39	AGGAGCACUAGGCGTGCAA
	P40	AGTGCTCCUGCAGCTAAAAAC
Y72F	P41	AGCTATAGCUCTTGCCATAC
	P42	AGCTATAGCUTTCTAATCAAAATTTATG
Y72W	P41	AGCTATAGCUCTTGCCATAC
	P43	AGCTATAGCUTGGTAATCAAAATTTATG
Y72A	P41	AGCTATAGCUCTTGCCATAC
	P44	AGCTATAGCUGCATAATCAAAATTTATG
A2D	P45	GGAGCACTAGGCGTGCAA
	P47	ATCAACTAAAAACATGGTGAGTGCC
A2I	P45	GGAGCACTAGGCGTGCAA
	P48	AATAACTAAAAACATGGTGAGTGCC
A2W	P45	GGAGCACTAGGCGTGCAA
	P49	CCAAACTAAAAACATGGTGAGTGCC
V1D	P45	GGAGCACTAGGCGTGCAA
	P52	TGCATCTAAAAACATGGTGAGTGCCTC
V1K	P45	GGAGCACTAGGCGTGCAA
	P53	TGCTTTTAAAAACATGGTGAGTGCCTCC
V1W	P45	GGAGCACTAGGCGTGCAA
	P54	TGCCCATAAAAACATGGTGAGTGCCTCC
Y69D	P56	TAAGCTTTCTTTGAACCAAAATTAGAAAACCAAG
	P57	ATCAGCTATAGCTCTTGCCATACTTTG
Y69K	P56	TAAGCTTTCTTTGAACCAAAATTAGAAAACCAAG
	P58	TTTAGCTATAGCTCTTGCCATACTTTG
Y69V	P56	TAAGCTTTCTTTGAACCAAAATTAGAAAACCAAG
	P59	AACAGCTATAGCTCTTGCCATACTTTG
A68D	P56	TAAGCTTTCTTTGAACCAAAATTAGAAAACCAAG
	P60	GTAATCTATAGCTCTTGCCATACTTTGCT
A68I	P56	TAAGCTTTCTTTGAACCAAAATTAGAAAACCAAG
	P61	GTAAATTATAGCTCTTGCCATACTTTGCT

S12 (Table S3). Amino acid sequences of the characterized subgroup I circular bacteriocins

Bacteriocin	Amino acid sequence
**Circularin_A**	VAGALGVQTAAATTIVNVILNAGTLVTVLGIIASIASGGAGTLMTIGWATFKATVQKLAKQSMARAIAY
**Cerecyclin**	VVSKLGWTGINIGTANALIGALMTGSDIWTAISVAGLAFGGGIGTAISTIGRKAIMEMVEKVGKKKAAQW
**Lactocyclicin_Q**	LIDHLGAPRWAVDTILGAIAVGNLASWVLALVPGPGWAVKAGLATAAAIVKHQGKAAAAAW
**Leucocyclicin_Q**	LVNQLGISKSLANTILGAIAVGNLASWLLALVPGPGWATKAALATAETIVKHEGKAAAIAW
**Garvicin_ML**	LVATGMAAGVAKTIVNAVSAGMDIATALSLFSGAFTAAGGIMALIKKYAQKKLWKQLIAA
**Uberolysin**	LAGYTGIASGTAKKVVDAIDKGAAAFVIISIISTVISAGALGAVSASADFIILTVKNYISRNLKAQAVIW
**Carnocyclin_A**	LVAYGIAQGTAEKVVSLINAGLTVGSIISILGGVTVGLSGVFTAVKAAIAKQGIKKAIQL
**Bacicyclicin XIN-1**	LTGIGIGSGTAATIVNWIMWGMSAATILSLISGVASGGAWILAGAREALKAGGKKAAIAW
**Amylocyclicin**	LASTLGISTAAAKKAIDIIDAASTIASIISLIGIVTGAGAISYAIVATAKTMIKKYGKKYAAAW
**Amylocyclicin_CMW1**	LASTLGISAAAAKKAIDIIDAASTIASIISLIGIVTGAGAISYAIVATAKTMIKKYGKKYAAAW
**AS-48**	MAKEFGIPAAVAGTVLNVVEAGGWVTTIVSILTAVGSGGLSLLAAAGRESIKAYLKKEIKKKGKRAVIAW
**BacA**	MAKEFGIPAAVAGTVLNVVEAGGWVTTIVSILTAVGSGGLSLLAAAGRESIKAYLKKEIKKKGKRAVIAW
**Aureocyclicin_4185**	LTGLGIGTGMAATIINAISVGLSAATILSLISGVASGGAWVLAGAKQALKEGGKKAGIAF
**Enterocin_NKR-5-3B**	LTANLGISSYAAKKVIDIINTGSAVATIIALVTAVVGGGLITAGIVATAKSLIKKYGAKYAAAW
**Pumilarin**	LAKEFGIPGSVAAVVLNVVEAGGAVTTIVSILTAVGSGGLSLIAAAGKETIRQYLKNEIKKKGRKAVIAW
